# Prediction method of surface settlement of rectangular pipe jacking tunnel based on improved PSO-BP neural network

**DOI:** 10.1038/s41598-023-32189-0

**Published:** 2023-04-04

**Authors:** Da Hu, Yongjia Hu, Shun Yi, Xiaoqiang Liang, Yongsuo Li, Xian Yang

**Affiliations:** 1 Hunan Engineering Research Center of Structural Safety and Disaster Prevention for Urban Underground Infrastructure, Hunan City University, Yiyang, 413000 People’s Republic of China; 2 College of Civil Engineering, Hunan City University, Yiyang, 413000 People’s Republic of China; 3 Hunan Provincial Key Laboratory of Key Technology on Hydropower Development, Power China Zhongnan Engineering Co. Ltd., Changsha, 410014 People’s Republic of China

**Keywords:** Engineering, Mathematics and computing

## Abstract

To provide theoretical support for the safety control of rectangular pipe jacking tunnels crossing an existing expressway, a method for predicting the surface settlement of a rectangular pipe jacking tunnel is proposed in this study. Therefore, based on the high approximation of the BP neural network to any function under the multiparameter input, the PSO-BP mixed prediction model of the ground subsidence of the ultrashallow buried large section rectangular pipe jacking tunnel is established by taking into account the adaptive mutation method, adopting the improved particle swarm optimization (IPSO) algorithm with adaptive inertia weight and mutation particles in the later stage to determine the optimal hyperparameters of the prediction model. Through the case study of an ultrashallow large cross-section rectangular pipe jacking tunnel, this algorithm is compared with the traditional algorithm and combined with field monitoring data for analysis and prediction. The prediction results show that compared with the traditional BP neural network prediction model, AWPSO-BP model and PWPSO-BP model, the improved PSO-BP mixed prediction model shows a more stable prediction effect when the change in surface subsidence is gentle and the concavity and convexity are large. The predicted subsidence value is close to the actual value, and the accuracy and robustness of the prediction are significantly improved.

## Introduction

Rectangular pipe jacking construction technology is often used in urban underpass tunnel projects because of its safe and reliable construction and low disturbance to the surrounding environment. As an increasing number of pipe jacking tunnels need to penetrate existing buildings (structures) closely, ensuring the stability of pipe jacking construction and reducing the impact of tunnel construction on existing buildings (structures) are hot issues at present. During the jacking process, the ultrashallow buried large-section rectangular pipe jacking tunnel inevitably disturbs the surrounding strata, changes the distribution of the stress field and displacement field of the strata, and finally affects the overall consolidation and settlement of the tunnel location with increasing time. The cause of formation deformation caused by pipe jacking construction is mainly that the formation damage disturbance caused by excavation cannot be compensated for in time, it is difficult to return to the initial state of undamaged disturbance, and a new three-dimensional equilibrium state can only be achieved through the necessary stress adjustment or change process. Therefore, it is of great significance to accurately predict the ground subsidence caused by rectangular pipe jacking tunnels to reduce the impact of engineering construction on the surrounding environment and ensure the safety of the project.

The existing surface settlement prediction methods mainly include empirical methods, analytical methods, numerical simulation methods, model test methods and artificial intelligence algorithm predictions. The well-known Peck formula in the empirical method^1–4^ can describe the general morphology of surface settlement and roughly believes that the lateral distribution of surface settlement generated by tunnel construction is approximately the normal distribution curve, but due to the large difference in the values of different strata empirical parameters, the two key parameters surface settlement trough width $i$ and maximum settlement $S_{\max }$ are related to many factors. Therefore, the empirical method expects settlement to have a wide distribution and cannot guarantee the accuracy and consistency of the prediction results. The analytical method^5–9^ often only considers the three factors of the frontal additional thrust of the pipe jacking tunnel, the friction between the pipe section and the soil mass and the soil loss, and it is difficult to consider the soil loss distribution and grouting situation. The calculation process is cumbersome and complex, which is difficult to use in actual engineering. The numerical simulation method^10–13^ can comprehensively consider the nature of the strata, the interaction between the pipe jacking machine and the strata, tunnel parameters, the location of the excavation surface, the tunnel lining and other factors, which provides a powerful visualization method for researchers to understand the failure mechanism inside the rock and soil mass. However, numerical simulation is intensive, laborious and laborious, and it is difficult to select geotechnical parameters and boundary conditions during the modelling process, which makes the analysis data different and uncertain. The model test^14–17^ method is widely used to study the evolution mechanism of settlement during pipe jacking construction, but the factors considered are limited and costly, so it cannot be used to predict surface settlement in real time. Moreover, due to the difference between the model test conditions and the actual engineering boundary conditions (such as the pipe jacking machine, size effect, etc.), the correction coefficient may have errors in the actual situation, and the conclusion needs to be verified by actual engineering.

Artificial intelligence algorithms have powerful information processing capabilities, such as nonlinearity, high parallelism and high fault tolerance learning and generalization capabilities^18^. Among them, the machine learning algorithm can learn the characteristics of the data through a sufficient number of sample inputs and then perform regression fitting analysis on the data to effectively analyse new inputs with similar patterns and make predictions^19^. By establishing a machine learning probability and statistics model, the internal connections of the input data can be automatically analysed, and the high-dimensional fitting of cross-cell and cross-latitude multiparameters can be realized. Moreover, the machine learning settlement prediction model is trained with fast calculation and accurate results, which meets the requirements of actual engineering to obtain surface settlement in a timely and accurate manner. Since the 1980s, some scholars have tried to use methods related to machine learning to solve practical tunnel engineering problems^20–28^. In recent years, artificial neural networks, support vector machines and random forest algorithms have become the main machine learning algorithms used to predict surface subsidence caused by shield tunnels. Ramezanshirazi^29^ and Tang^30^ used machine learning algorithms to effectively predict surface subsidence caused by tunnel construction. Chen et al.^31^ found that regression neural networks and random forest algorithms can perform best among the six machine learning algorithms and accurately identify the evolution of settlement caused by tunnels. Elbaz et al.^32^ established an improved particle swarm optimization algorithm (PSO) combined with an adaptive neural fuzzy inference system (ANFIS) based on the fuzzy C-means (FCM) clustering method for the prediction of the shield performance of metro tunnels. Cao et al.^33^ proposed a fully ensembled empirical model with adaptive noise length and short memory to monitor ground subsidence during tunnel construction, which can divide one-dimensional data into multidimensional data. It can be seen from the above that in the past tunnel surface settlement prediction problem, most of them chose to use several single algorithms for separate prediction, and the results were compared to illustrate the superiority of a certain algorithm.

To improve the prediction accuracy, this paper intends to use the combination of the two algorithms to build a predictive model to improve the prediction accuracy and robustness. In this paper, an improved particle swarm algorithm is proposed to balance the local and global optimization ability of particles and enhance the ability to jump out of a local minimum in the late stage of particle search by adaptive inertial weights and the position of mutated particles. Relying on the real-time monitoring data of rectangular pipe jacking tunnel construction on the west extension of Liuye Avenue, the improved particle swarm algorithm (IPSO) combined with the traditional BP neural network is used to establish a PSO-BP hybrid prediction model for the surface settlement of rectangular pipe jacking tunnels, and the prediction performance and stability of the old and new algorithm models are compared and analysed to provide theoretical support and technical reference for the design and construction of similar rectangular pipe jacking tunnels. In addition, improved particle swarm optimization (IPSO) has been used successfully in other fields. For example, Jin et al.^34^ built the PSO-BP neural network, wrote the PSO-BP code, collected the historical container throughput data of Shenzhen Port, set the relevant parameters, and used the PSO-BP model to predict the container throughput of Shenzhen Port. Wan et al.^35^ proposed a BP neural network adaptive PID controller based on particle swarm optimization (PSO). Using the Chinese national standard variable rate fertilizer seeder control system, the system's performance indicators, such as fertilizer rate adjustment range, fertilizer rate adjustment response time and fertilizer rate control accuracy, were evaluated, and the variable rate fertilizer performance parameters were successfully improved. Shu et al.^36^ proposed a PSO-BP neural network to evaluate the customer perceived value of new Chinese clothing. The experimental results show that the PSO-BP neural network can accurately evaluate the customer perceived value of the new Chinese style clothing, and enterprises can improve the product design quality through the evaluation index and model of the customer perceived value of the new Chinese style clothing, thereby improving the sustainable competitive advantage of enterprises and finally realizing the sustainable development of the new Chinese style clothing industry.

## Settlement prediction methods

### BP neural network algorithm

**Figure 1:**
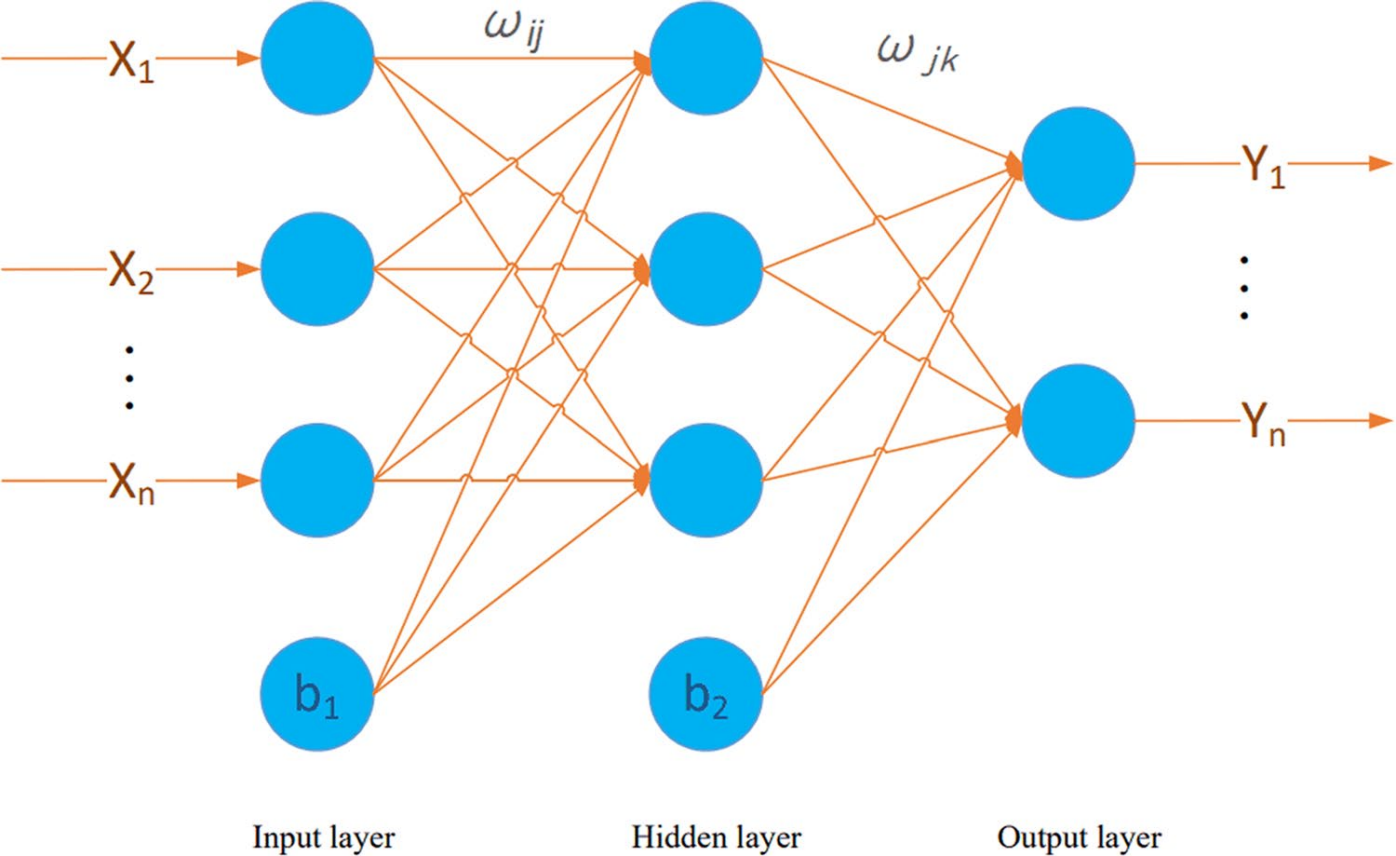
Neural network topology diagram.

The BP neural network (backpropagation neural network), also known as the error backpropagation neural network, contains two learning processes of forwards transmission of input signals and backpropagation of error signals, mainly composed of an input layer, hidden layer, and output layer in structure. Its topology is shown in Fig. 1. Data are obtained from the input layer $\left\{ {x_{m} ,m = 1,2,3, \ldots n} \right\}$, and the output value of the layer $x_{i}$ is obtained through formula (1). To facilitate the operation, no activation operation is carried out in the output layer. At the same time, the obtained output value is backpropagated by error, the weight and threshold between each layer are adjusted, and the prediction accuracy of the neural network is improved through continuous forwards and negative propagation until the error value is less than the target value and the best model is obtained. The number of layers and number of hidden layer neurons are very important for the accuracy of the model. The number of nodes is small, the learning ability of the neural network is low, the accuracy rate is low, the number of nodes is large, the network is prone to overfitting, and the calculation time is long.

1 $$x_{i} = f\left( {\sum\limits_{k = 0}^{n - 1} {\omega_{ij} } x_{{\text{m}}} - b_{ij} } \right)$$

Formula:$\omega_{ij}$ is the weight of the node,$b_{ij}$ is the threshold value of the point

2 $$f\left( x \right) = \frac{1}{{1 + e^{ - x} }}$$

$f$ is the activation function, and the activation function of the hidden layer in this article uses the $sigmoid$ function (as shown in Eq. (2)) for convenient operation, and the output layer does not perform activation calculation.

### Particle swarm algorithm

The particle swarm algorithm is an evolutionary computing technique that simulates a bird in a flock by designing a massless particle with only two properties: speed and position, where speed represents how fast it moves, and position represents the direction of movement. Each particle in the search space of the optimal solution is recorded as the current individual extreme value, and the individual extreme value is shared with other particles in the entire particle group. The optimal individual extreme value is found as the current global optimal solution of the entire particle group. All particles in the particle group adjust their speed and position according to the current individual extreme value they find and the current global optimal solution shared by the entire particle group and at the same time continue to iterate until the optimal solution of the target is found. The speed and position of the particles are set as follows. The formula for updating the velocity and position of the particles is shown in Eq. (3):

3 $$\begin{gathered} v_{i,j}^{k + 1} = \omega v_{i,j}^{k} + c_{1} r_{1} \left( {p_{i,j}^{k} - x_{i,j}^{k} } \right) + c_{2} r_{2} \left( {g_{i,j}^{k} - x_{i,j}^{k} } \right) \hfill \\ x_{i,j}^{k + 1} = x_{i,j}^{k} + v_{i,j}^{k + 1} \hfill \\ \end{gathered}$$

Formula: k is the number of iterations;$\omega$ is the inertia weight;$c_{1} ,c_{2}$ are the "individual" and "global" learning factors;$r_{1} ,r_{2}$ is the interval [0,1] random numbers; $p_{i,j} ,g_{i,j}$ are the individual optimal and global optimal solutions of particle $i$ in dimension $j$, respectively.

### PSO-BP hybrid predictive model

**Figure 2:**
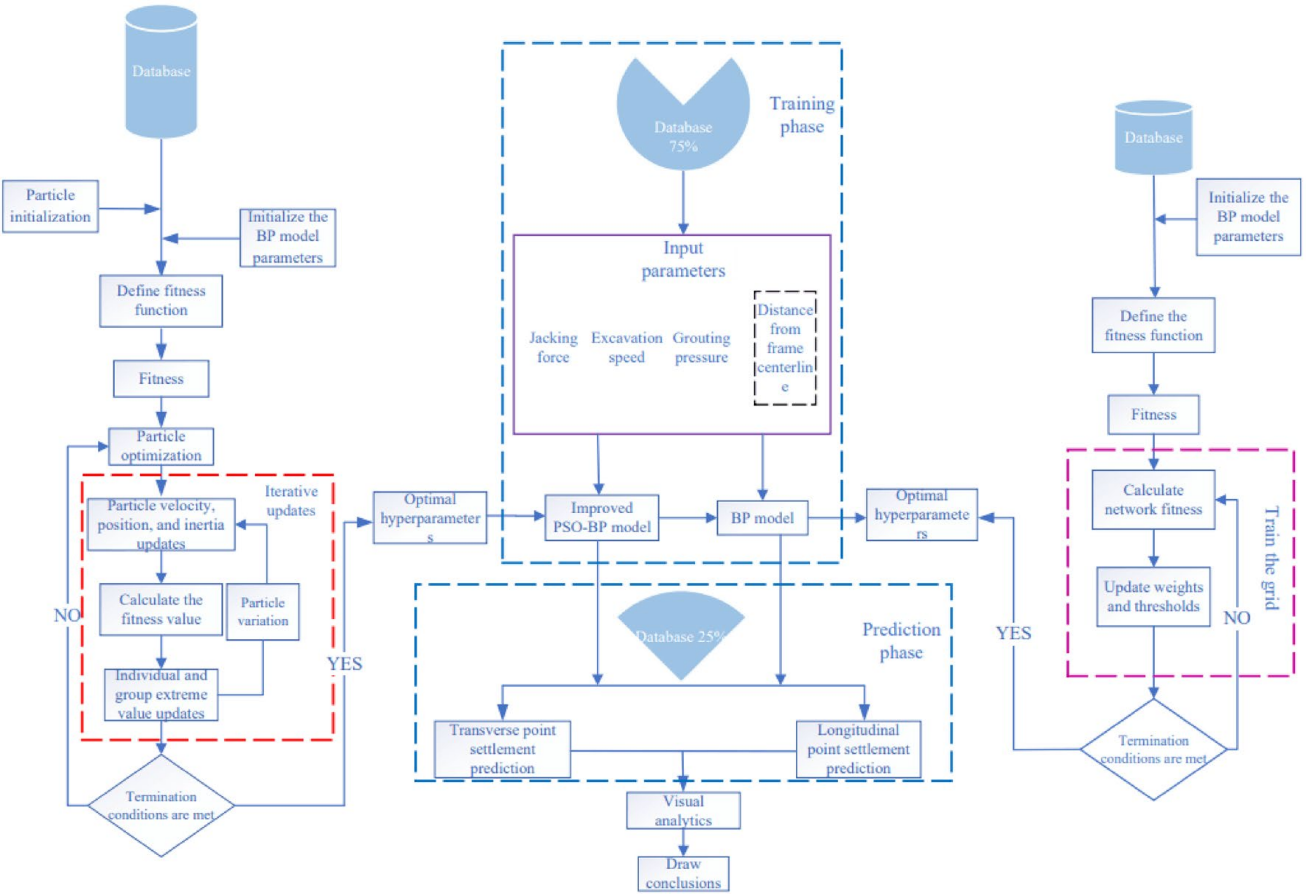
Model framework and step flow chart.

The improved PSO-BP neural network model proposed in this paper is hereinafter collectively referred to as the IPSO-BP hybrid prediction model, and the outstanding advantage of the traditional BP neural network is that it has a strong nonlinear mapping ability and flexible network structure. The number of intermediate layers and the number of neurons in each layer can be set arbitrarily according to the specific situation, and the operation is relatively simple and convenient, but the learning rate is slow, the convergence time is long, and the random initial weights and thresholds will have a greater impact on the results, thereby reducing the prediction performance of the network as a whole. To reduce the impact, this paper adopts the improved particle swarm algorithm and introduces the linear decreasing inertia weight $\omega^{i}$, which better balances the global search ability and local search ability of the algorithm, and the $\omega^{i}$ update formula is shown in (4). Through the global search of particles, when the later particle search falls into the local optimum, the mutation jumps out of the local point and studies until the optimal individual in Eq. (5) and the loss target value are found, and the fitness function fitness is shown in Eq. (6); otherwise, it continues to iterate to optimize the adjustment of weights and thresholds to accelerate the convergence speed of the network and improve the learning efficiency of the network. The specific calculation process and model framework diagram are shown in Fig. 2. To further reflect the superiority of the improved method in this paper, this paper adds an improved standard PSO-BP model based on formula (4) (AWPSO-BP) and an improved standard PSO-BP model based on formula (5) (PWPSO-BP) for comparative analysis:

4 $$\omega^{i} = \omega_{start} - \left( {\omega_{start} - \omega_{end} } \right) \cdot \left( {{\raise0.7ex\hbox{$i$} \!\mathord{\left/ {\vphantom {i {i_{all} }}}\right.\kern-0pt} \!\lower0.7ex\hbox{${i_{all} }$}}} \right)$$

5 $$x_{i,j}^{k + 1} = Crx_{i,j}^{k}$$

6 $$F{\text{itness = }}\sum\limits_{i = 1}^{n} {\left| {x_{i} - y_{i} } \right|}$$

Formula:$\omega_{start}$ and $\omega_{end}$ are the starting inertia weights;$i$ and $i_{all}$ are the current number of iterations and the total number of iterations; *C* = 0.95, *r* is [0, 1] random number;$x_{i}$ and $y_{i}$ are output layers, respectively Predicted and true values.

### Data processing and model evaluation

To avoid the phenomenon of gradient explosion in the network training process due to the different magnitudes of each index in the calculation process, the data should be preprocessed after the normalization operation is entered into the grid, and the normalization formula is shown in Eq. (7), mapping all data to the interval of [− 1,1] and inverse-normalizing and visualizing the results after all calculations are completed.

7 $$x_{m} = 2 \times \frac{{x - x_{\min } }}{{x_{\max } - x_{\min } }} - 1$$

Formula:$x_{\min }$ and $x_{\max }$ are the maximum values of $x$.

To evaluate the performance difference between the two algorithms in predicting the settlement of tunnel construction strata excavated by the rectangular pipe jacking method, three error indexes are used to evaluate the superiority of the model by using three error indexes: correlation coefficient (R), mean squared error (MSE), and mean absolute error (MAE), such as Eqs. (8)–(10).

8 $$R^{{}} = \sqrt {1 - \frac{{\sum\limits_{i = 1}^{n} {\left( {y_{i} - f_{i} } \right)^{2} } }}{{\sum\limits_{i = 1}^{n} {\left( {y_{i} - \tilde{y}} \right)^{2} } }}}$$

9 $$MSE = \frac{{\sum\limits_{i = 1}^{n} {\left( {y_{i} - f_{i} } \right)^{2} } }}{N}$$

10 $$MAE = \frac{{\sum\limits_{i = 1}^{n} {\left| {f_{i} - y_{i} } \right|} }}{N}$$

Formula: *y*_*i*_ is the true value of the data; *f*_*i*_ is the predicted value; ${\tilde{\text{y}}}$ is the average; N is the total. A larger $R^{{}}$ indicates a higher correlation between the independent variable and the surface settlement of the dependent variable. The smaller the values of $MSE$ and $MAE$ are, the lower the error of the model's prediction and the better the overall performance.

### Algorithm parameter initialization

The number of hidden layer nodes is set according to Kolmogorov's theorem, $s = 2n + 1$ ($n$ is the number of input layer nodes, and $s$ is the number of hidden layer nodes). Table 1 shows the remaining hyperparameter settings:

**Table 1:** Superparameter settings.

Algorithm	Hyperparameter type	Value
BP	Hidden layer nodes	9
	Learning rate	0.001
	Network target loss value	0.0004
	Number of learnings	2000
PSO	Group size	20
	Inertia weights $\omega_{start}$ and $\omega_{end}$	0.9 ;0.4
	Learning factor $c_{1}$ and $c_{2}$	1.49;1.49
	Speed extremes	1.3; − 1.3
	Total number of iterations	100
	Optimize the target loss value	0.0001

## Project overview

### Case introduction

**Figure 3:**
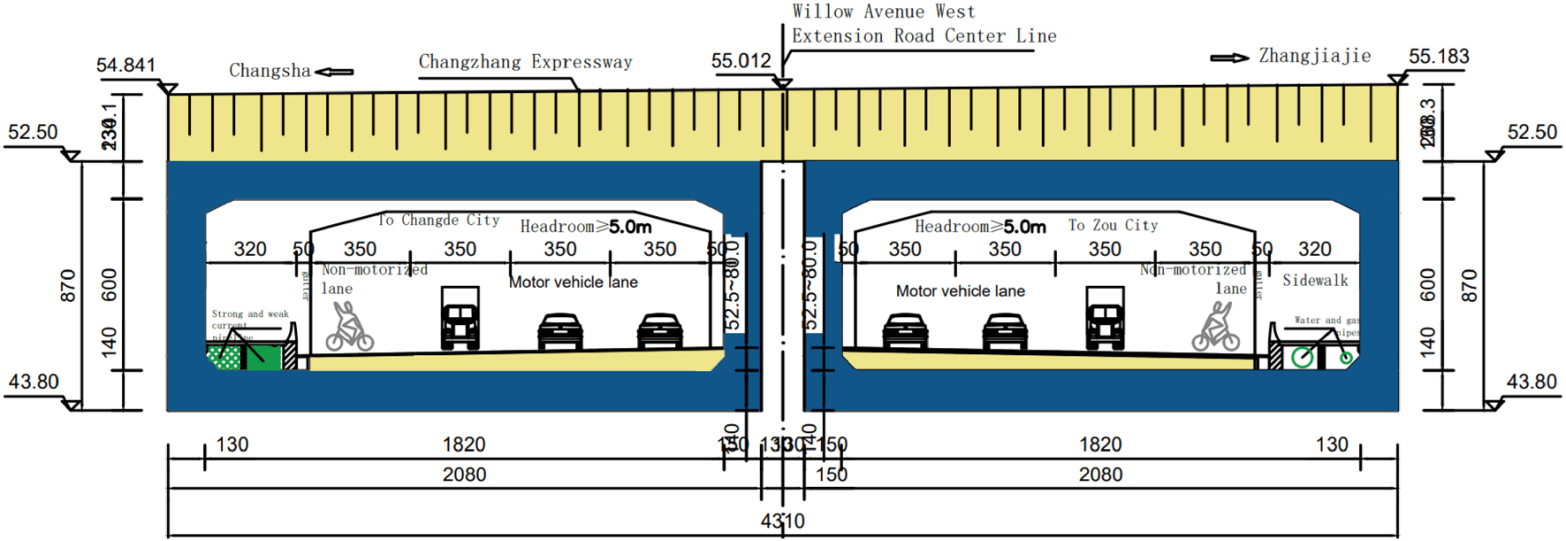
Schematic diagram of the rectangular pipe jacking tunnel of the west extension line of LiuYe Avenue.

At the central pile number K7 + 081.113 in the center of the road, the west extension of Liuye Avenue is equipped with a two-hole net aperture of 18.2 × 6.0 m diagonal rectangular pipe jacking tunnel, the rectangular pipe jacking tunnel and the long-tension high-speed diagonal intersection of 63.881°, the total length of each hole rectangular pipe jacking tunnel is 51 m, and the thickness of the tunnel top cover is approximately 2.000 ~ 2.683 m. The rectangular pipe jacking tunnel is prefabricated in 4 sections, each with a length of 12 m, 13 m, 13 m, and 13 m. The tunnel construction adopts the form of setting up working pits, back walls, skateboards, and steel shield supports on site and adopts the construction process of "diagonal, diagonal, and frontal roof". The settlement joints connected by each rectangular tube jacking tunnel are 4 cm wide. The general arrangement of the rectangular pipe jacking tunnel of the west extension of Willow Avenue is shown in Fig. 3.

**Figure 4:**
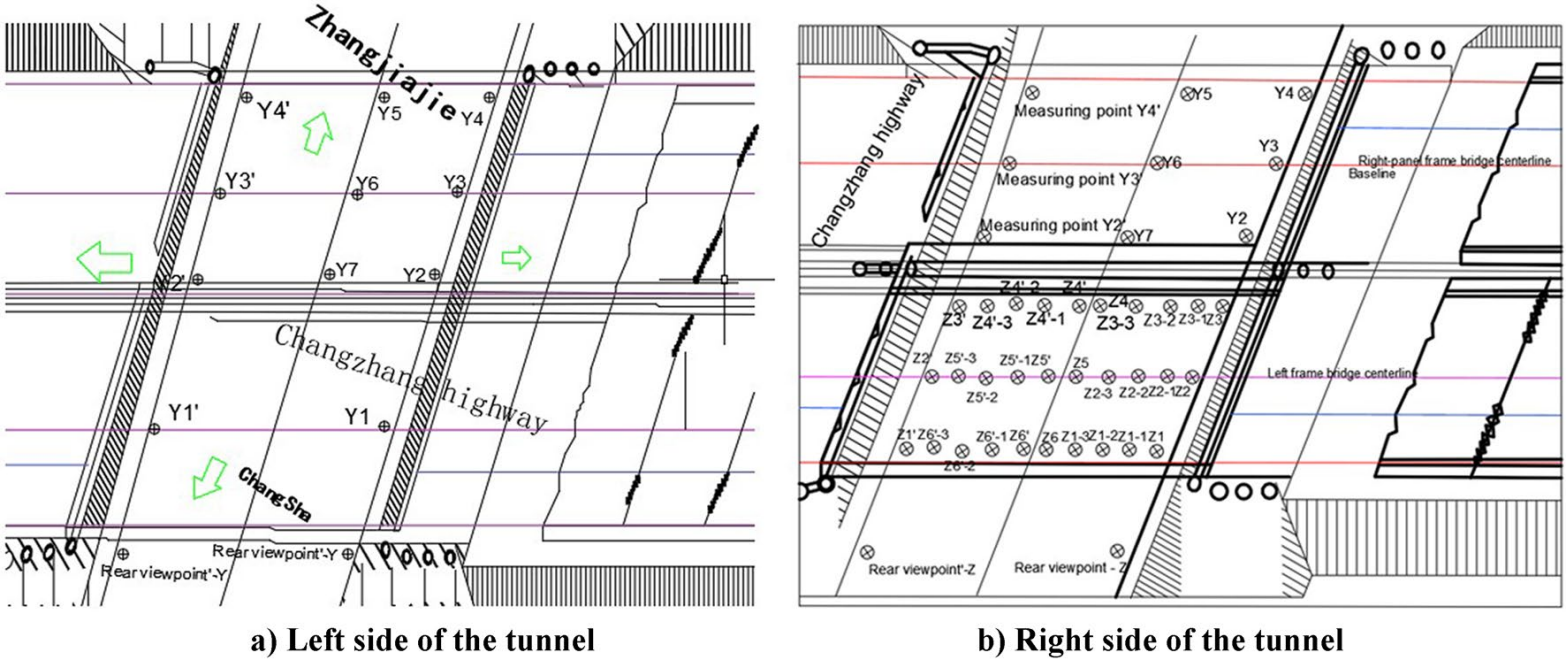
Layout of pavement deformation observation points.

In the construction of this project, it is impossible to interrupt the traffic on the Changzhang Expressway, and the steel shield bracket is erected on site. A matching jack is laid in the relay room and tail end of the rectangular pipe jacking tunnel for jacking construction, but the jacking construction will disturb the highway subgrade and cause the road surface to settle and deform, which easily affects driving safety. To this end, steel shield brackets are used to maintain the slope ratio of the highway, which improves the safety and applicability of the construction, especially for tunnel jacking construction, which is more reliable and minimizes the repeated disturbance of the highway subgrade during shallow cladding jacking construction. However, the steepening rate of the rectangular pipe jacking tunnel is too fast, and the core soil is overexcavated, which will still cause the road surface to sink. In addition, when the rectangular pipe jacking tunnel comes out of the hole, due to the thin overlying soil layer (only 2 m at the thinnest point of this project), if the jacking rate is too fast, the jacking rectangular pipe jacking tunnel will drive the upper layer of overburden to move along the top direction (backsoil effect), resulting in horizontal displacement of the door frame beam at the exit opening and even causing longitudinal cracking of the road surface. The three-dimensional measurement point layout of the road surface is shown in Fig. 4a,b.

### Database

All the data in this article are derived from the construction site monitoring of the rectangular pipe jacking tunnel on Willow Leaf Avenue. According to the research of Zhang et al., the factors affecting surface settlement can be divided into four categories: tunnel geometric parameters, excavation parameters, geological conditions and abnormal factors. Since the jacking construction is excavated at the same level, the relevant specifications and burial depth of the tunnel hardly change, and the influence of the tunnel geometry on the results is not taken into account here. In the selection of excavation parameters, the jacking force and excavation speed are mainly selected, which are the key parameters of pipe jacking construction control and can be collected in real time through sensors and other data acquisition systems, which are accurate and reliable. At the same time, it also takes into account the large influence of parameters such as grouting pressure on overcutout voids and later settlement. Therefore, several variables of the above boring parameters are the main factors affecting surface settlement. The geological conditions need to consider the basic physical and mechanical properties of the soil layer and the spatial position of the soil layer, and the soil excavated in this project is relatively homogeneous, all silty clay, so the influence of geological conditions on the results can be ignored. Since tunnel jacking construction adopts the method of manual excavation, the process is relatively simple, and there are few abnormal factors, so it is not considered for the time being. In summary, based on the important and easy-to-obtain indicators in the actual construction process, the jacking force, tunneling speed, and grouting pressure are selected as the input parameters of the prediction model. The jacking force is a major factor causing soil settlement. The jacking speed directly affects the stability of the soil layer during the construction process. As an auxiliary process in the jacking process, the grouting pressure can increase the degree of friction in the jacking process and accelerate the construction progress, and a total of 27 sets of data were used, of which approximately 75% of the first 20 groups were used for the training and calculation of the model, and the last 7 groups were used for the prediction of the test set. The specific measurement point data sets are shown in Tables 2 and 3:

**Table 2:** Data set of lateral measuring points.

Variable	Parameter type	Data (26 sets)			
		Min	Max	Ave	S.D
Left, top thrust (KN)	Input	15,780.31	47,755.17	34,566.32	9608.77
Right, advance speed (m/d)		0.825	1.985	1.640	0.223
Left, advance speed (m/d)		0.777	1.885	1.409	0.249
Grouting pressure (MPa)		0.063	0.416	0.271	0.079
Y1	Output	− 4	2	− 0.231	1.250
Y2		− 27	13	− 4.385	8.513
Y3		− 5.5	5	0.038	2.742
Z-1		− 10	1	− 2.385	2.573
Z-2		− 9	10	− 1.5	4.236
Z-3		− 8	44	− 1.654	2.674

**Table 3:** Data set of longitudinal measuring points.

Right portrait		Left portrait	
Distance from centerline/m	Sedimentation value/mm	Distance from centerline/m	Sedimentation value/mm
21.55	2	21.55	− 3
11.15	3	11.15	1
0.75	2	0.75	− 5
− 11.5	1.8	− 0.75	3
–	–	− 11.15	6
–	–	− 21.55	8.2

## Predicting the outcome

### Adaptability changes

**Figure 5:**
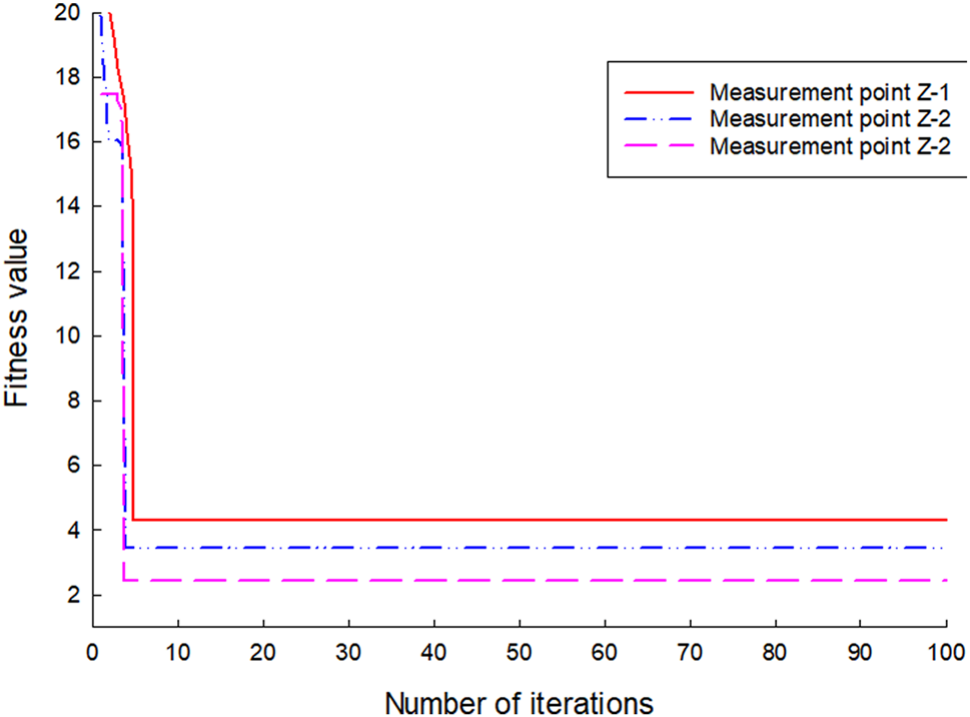
Fitness change diagram of the left measuring point.

**Figure 6:**
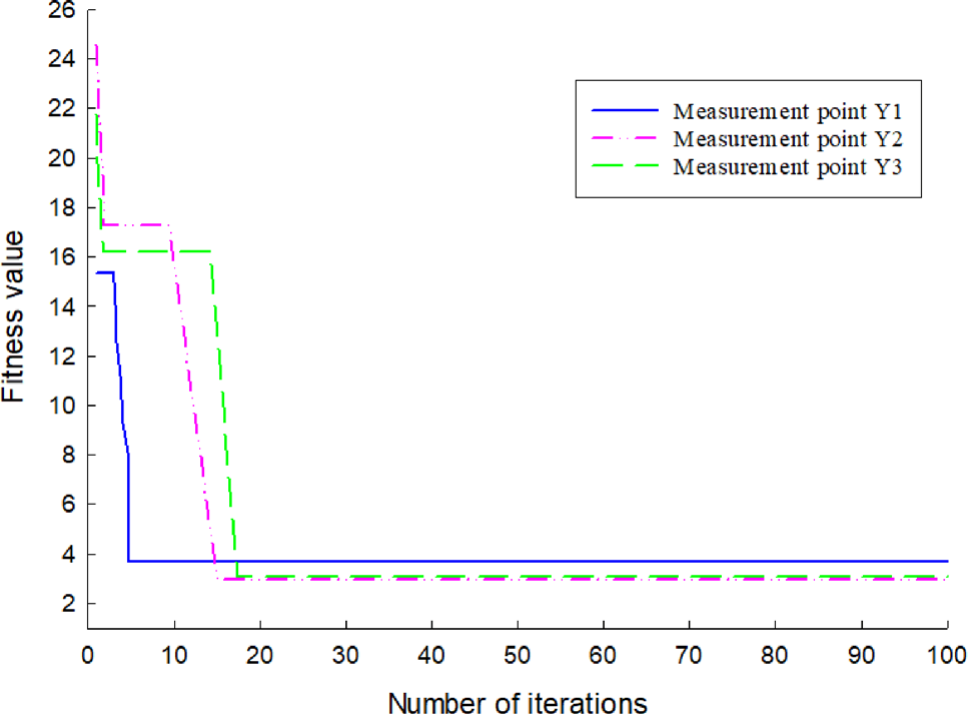
Fitness change diagram of the right measuring point.

As the grid is iteratively updated by backpropagation and the overall error is gradually reduced, Figs. 5 and 6 show the variation process of the optimal fitness value of the self-variant IPSO-BP algorithm for the lateral 26-day settlement data of the left and right rectangular pipe jacking tunnels Z-1, Z-2, Z-3, Y1, Y2 and Y3.

### Sedimentation data analysis

**Figure 7:**
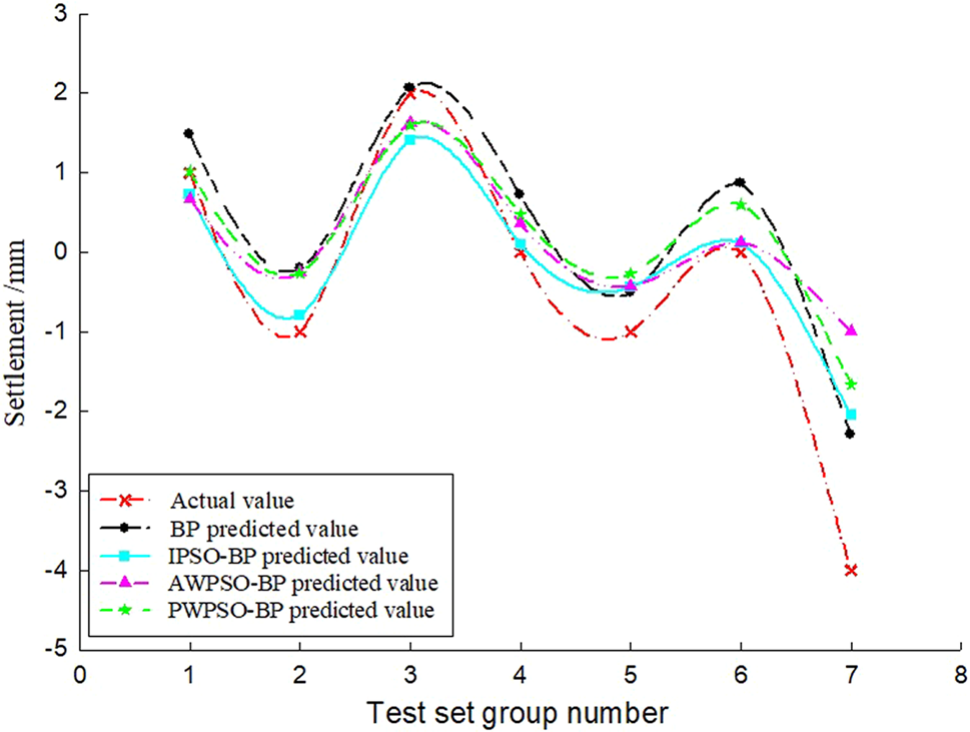
Right Y1 measuring point.

**Figure 8:**
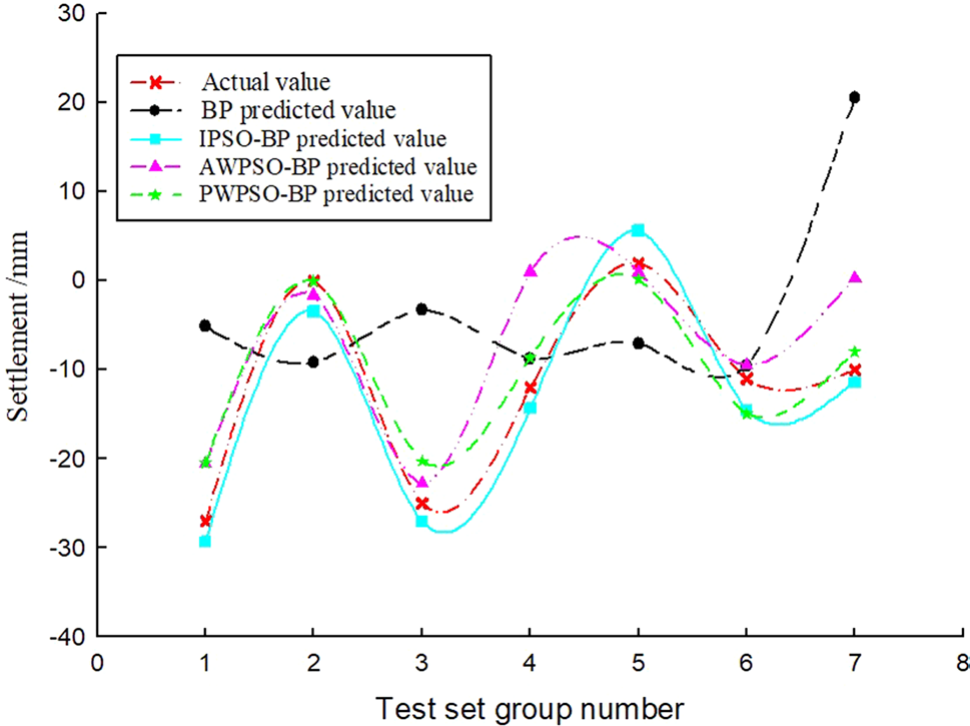
Right Y2 measuring point.

**Figure 9:**
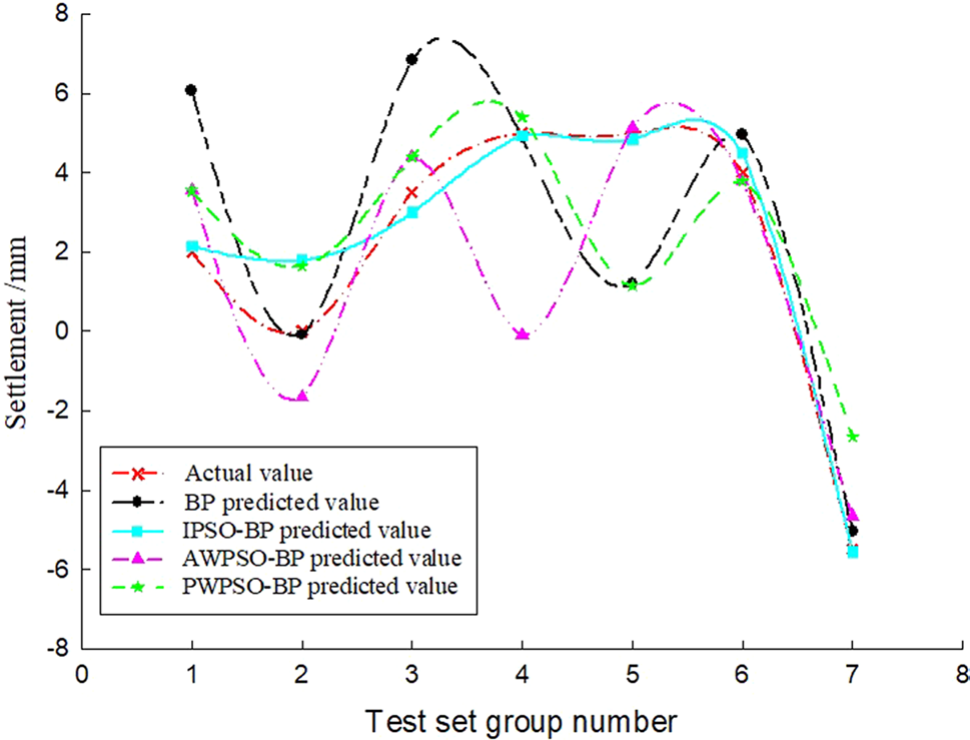
Right Y3 measuring point.

**Figure 10:**
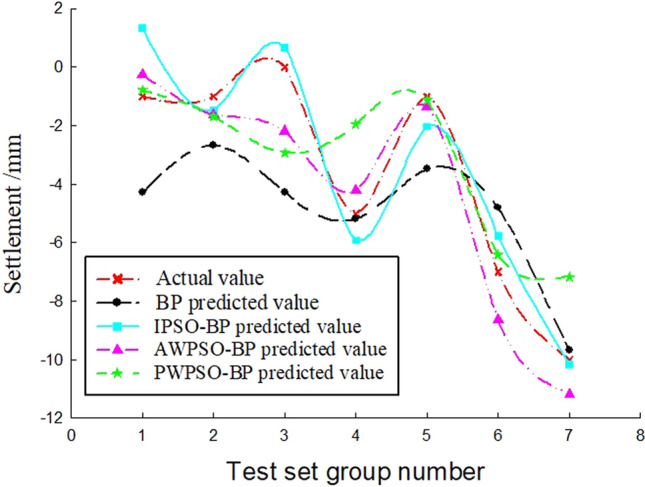
Left Z-1 measuring point.

**Figure 11:**
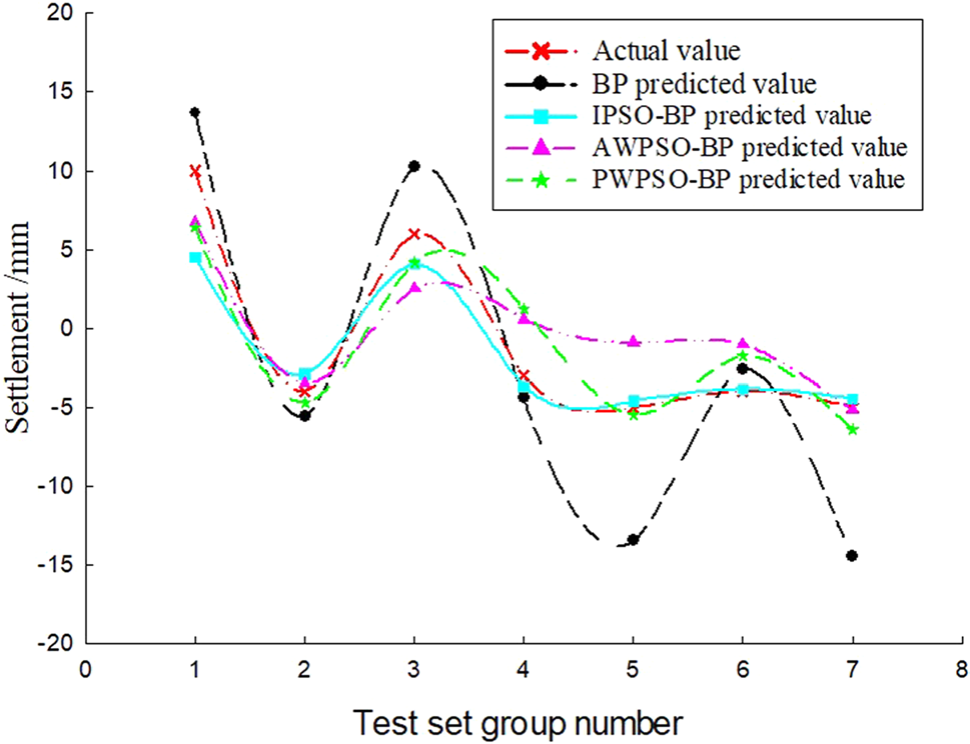
Left Z-2 measuring point.

**Figure 12:**
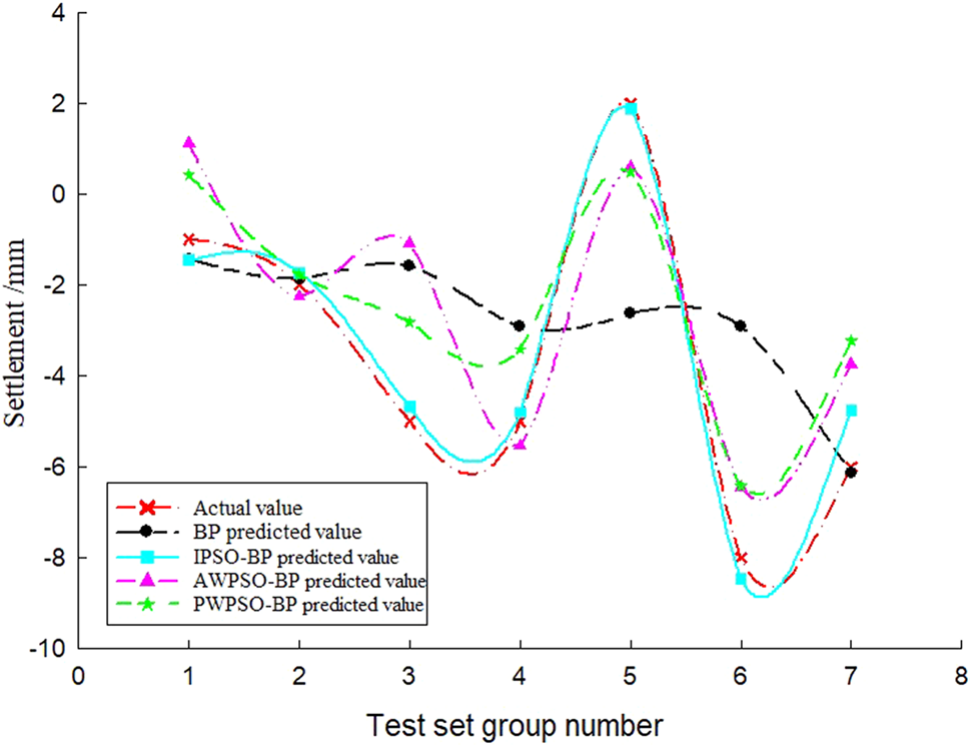
Left Z-3 measuring point.

To study the sedimentation change law of the measurement point with time, considering the influence of the jacking force, ejection speed and grouting pressure, the BP and IPSO-BP hybrid prediction model were constructed based on the monitoring data and model parameters. The test set of the corresponding measurement points on the left and right amplitudes of the rectangular pipe jacking tunnel is trained, and the prediction results and real settlement data are obtained as shown in Figs. 7, 8, 9, 10, 11 and 12.

Analysis of Figs. 7, 8, 9, 10, 11 and 12 shows that when the fluctuation value of the settlement value is large, the prediction effect of the traditional BP neural network is obviously poor; when the data fluctuate more gently, the forecast performance is average. The self-varying IPSO-BP hybrid prediction model is very close to the real settlement value of other points, except for the distortion of individual point data and poor prediction effect, and the comprehensive prediction effect is obviously better than that of the traditional BP neural network model.

**Figure 13:**
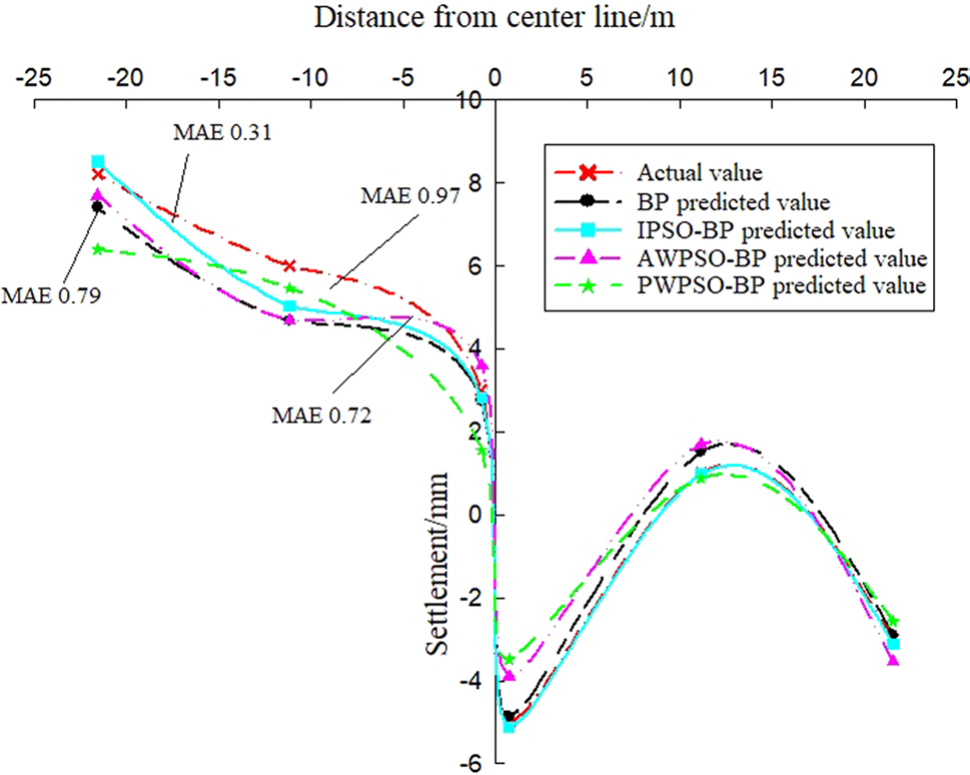
Longitudinal measuring points on the left.

**Figure 14:**
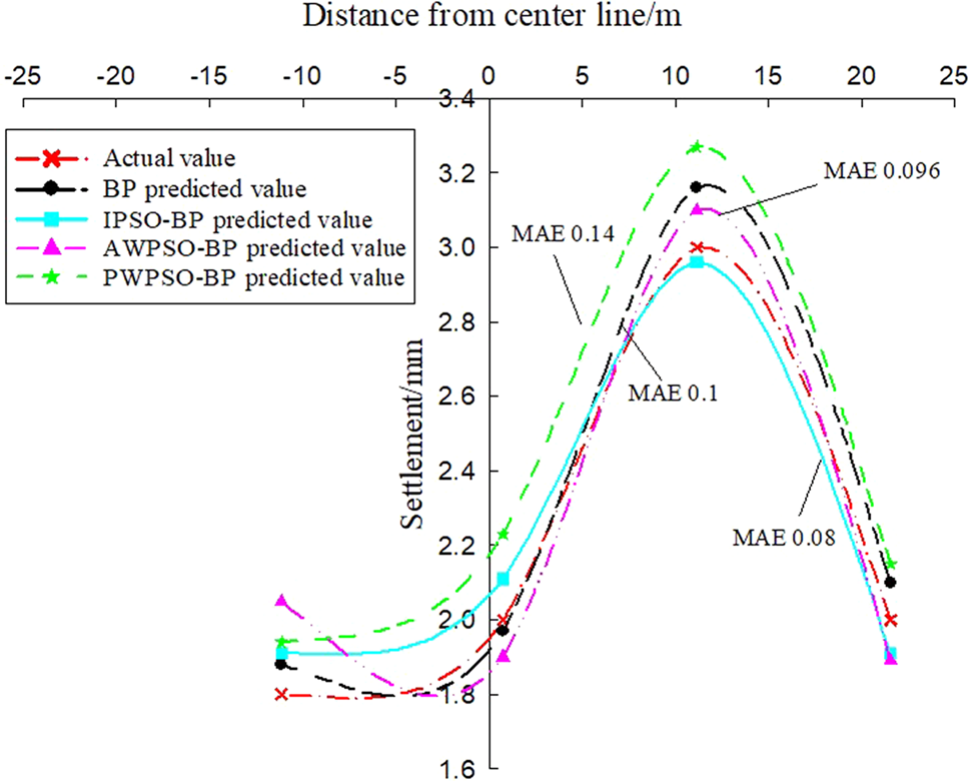
Longitudinal measuring points on the right.

**Figure 15:**
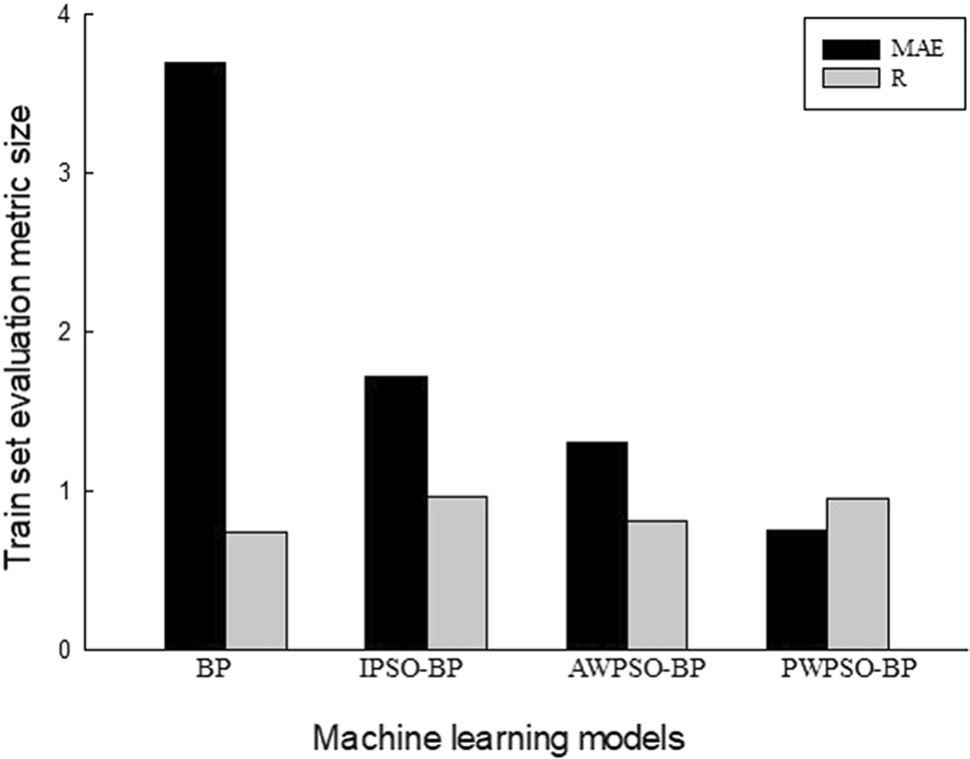
Performance evaluation index of the algorithm model of the right training set.

To study the settlement law of measurement points on the same section at the same time, the variables in the model can be regarded as constants, and the only independent variable is the distance of the measurement point from the centerline of the rectangular pipe jacking tunnel (the spatial position of the measurement point of the same section). According to the distribution of measurement points, the settlement of longitudinal measurement points is predicted and analysed, and the real settlement and predicted settlement pairs of the left and right amplitude Y4'-Z1' and Y4'-Y1' measurement points are obtained, as shown in Figs. 14 and 15. Figures 13 and 14 show that the prediction effect of the four algorithm models on the longitudinal settlement data is good, but the improved IPSO-BP hybrid prediction model has a lower error index, and the performance is more stable and superior.

### Algorithm performance comparison

**Figure 16:**
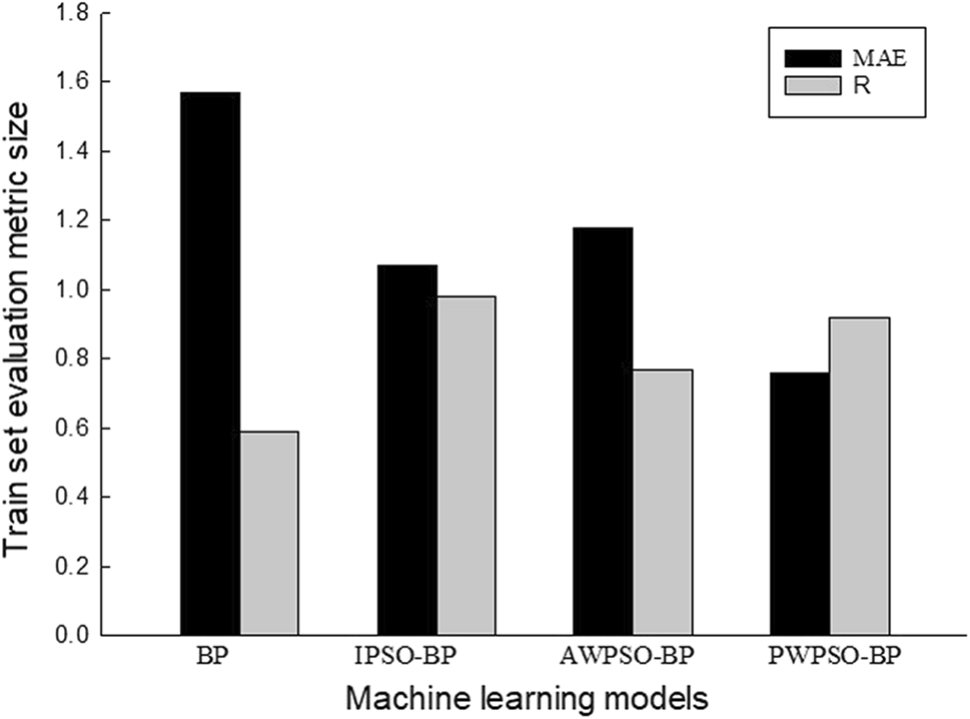
Performance evaluation index of the algorithm model of the left training set.

**Figure 17:**
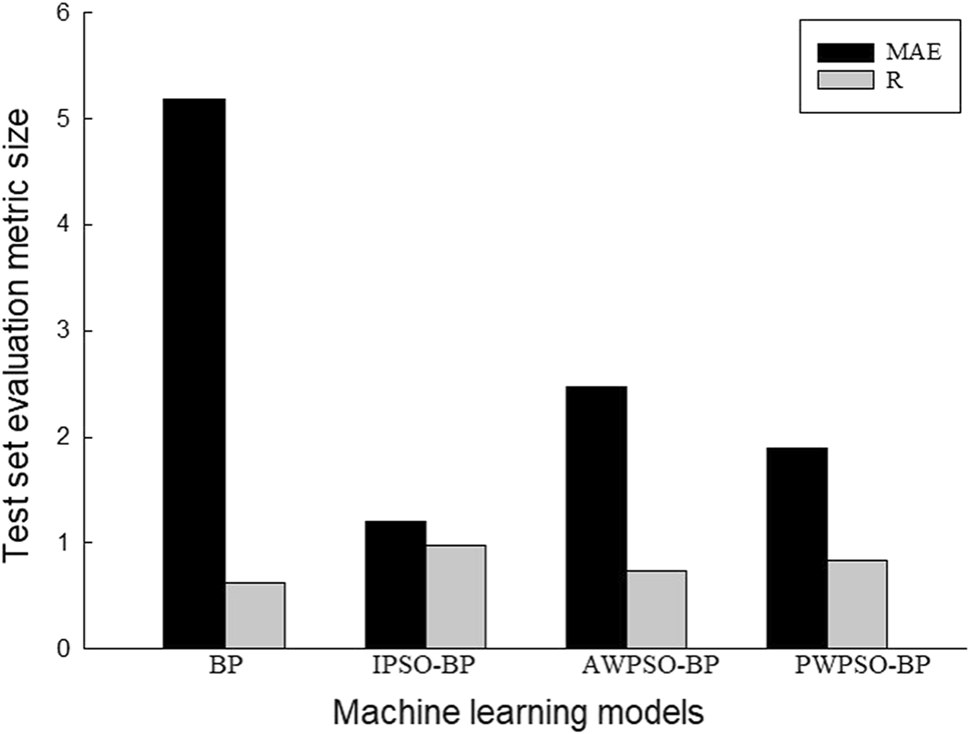
Performance evaluation index of the algorithm model of the right test set.

**Figure 18:**
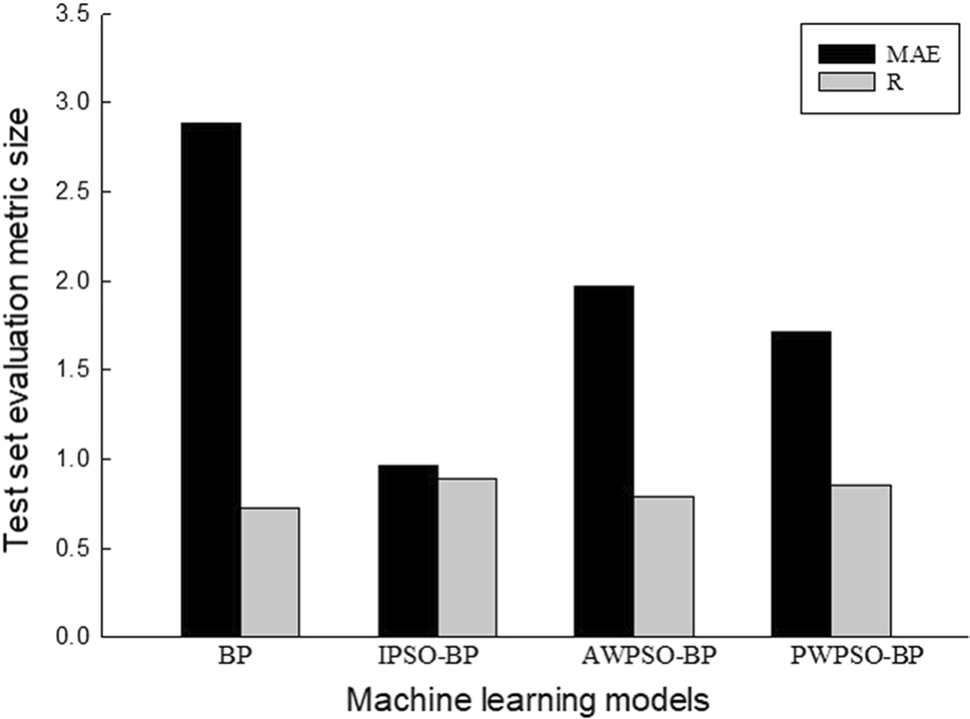
Performance evaluation index of the algorithm model of the left test set.

To better compare the prediction performance of the IPSO-BP hybrid prediction model, the average value of the left and right horizontal measurement point evaluation index is mainly used for comparison, and the prediction performance is shown in Figs. 15, 16, 17 and 18.

**Figure 19:**
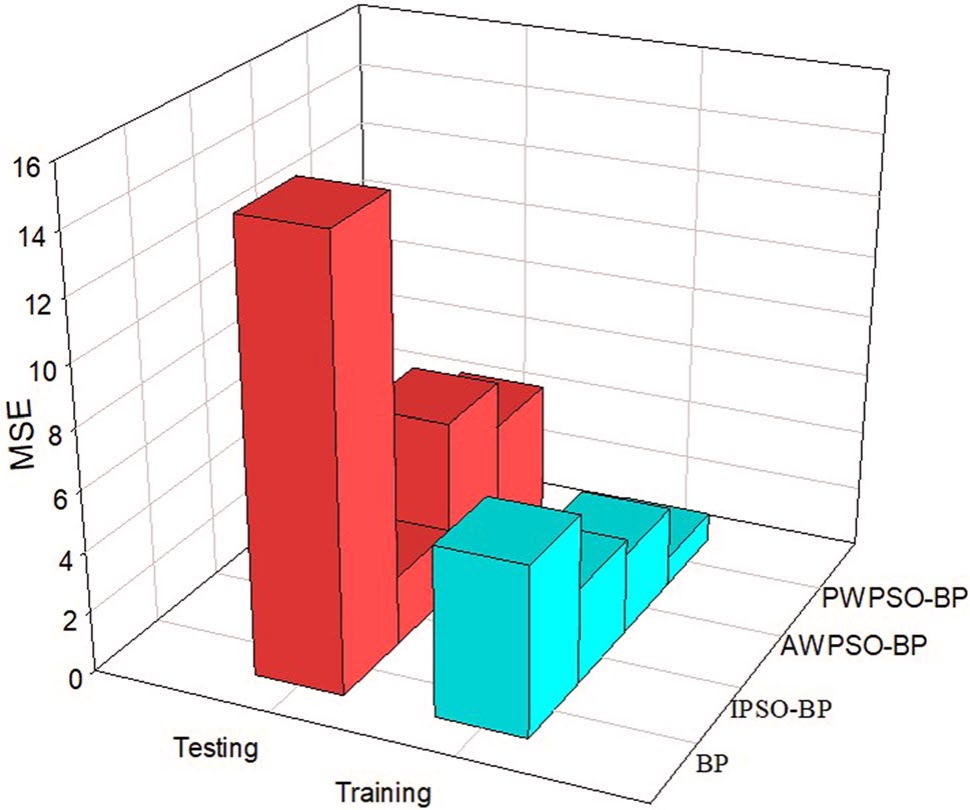
MSE comparison diagram of the left frame.

**Figure 20:**
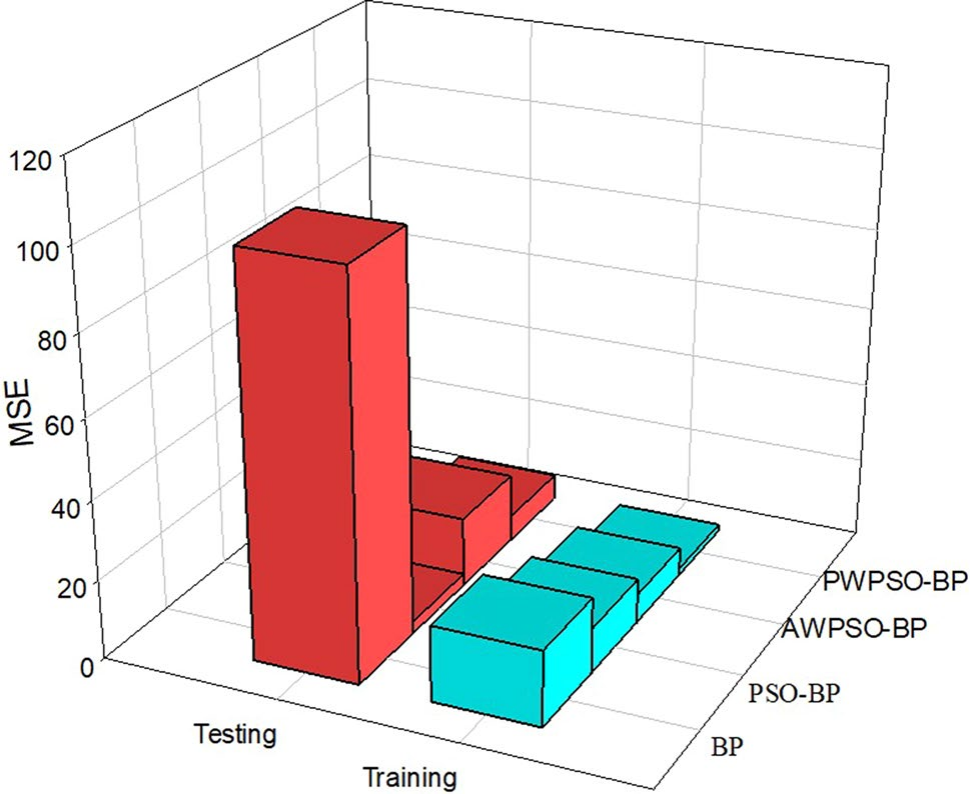
MSE comparison diagram of the right frame.

The comparative analysis of Figs. 15, 16, 17 and 18 shows that the PWPSO-BP model has the lowest MAE value of 0.75 and the highest R of 0.95 in the rectangular pipe jacking tunnel training set, followed by the IPSO-BP algorithm model. The lowest MAE value is 1.07, and the highest R is 0.98. The other two models have relatively high error indicators, indicating that their realization in the training set is relatively poor. In the test set, the IPSO-BP algorithm model has the lowest MAE value relative to the other three models, with a minimum difference of 0.71 and a maximum of 3.99. At the same time, the R value is the largest but not much different from the PWPSO-BP model. The R values of the other models are all above 0.6, and the effect is general. From Figs. 19 and 20, it can be seen that the overall MSE error of the IPSO-BP model is similar to that of PWPSO-BP, but the gap is not large and is better than other models. It is worth noting that the individual points with larger errors may be caused by not considering the surrounding uncertainties such as highway traffic load. In summary, the IPSO-BP algorithm in this example has deeper learning power and stability than the other three models, with faster convergence, an obvious optimization effect, and relatively short time consumption.

## Conclusion

In this paper, the improved IPSO-BP algorithm with adaptive inertial weights and particle variability factors is introduced, combined with the traditional BP neural network, and the horizontal and longitudinal surface settlement data of the corresponding monitoring points are compared and predicted. The main conclusions are as follows:

A) The improved adaptive weight can make the particles dynamically adjust with the iterative process, which greatly increases the probability of finding the optimal parameters. At the same time, adding the factor of particle mutation can ensure that the particles jump out of the local optimal value in the later stage of convergence to carry out a new optimization path. Theoretically, as long as the number of iterations is large, the algorithm for optimizing the optimal threshold and weight of the BP neural network introduced in this paper must be able to find the best parameters for model prediction. The results show that the improved IPSO-BP algorithm performs better than the other comparison models in this paper.
B) For transverse measuring points, the settlement prediction error of the improved IPSO-BP algorithm model is significantly smaller than that of the other three traditional algorithm models. The maximum single-point prediction error of the former is − 2 mm, the PWPSO-BP model is 6.57 mm, the AWPSO-BP model is 13.12 mm, and the BP model is 30 mm. The average prediction error of IPSO-BP is − 1.814 mm, that of PWPSO-BP is 4.28 mm, that of AWPSO-BP is 4.33 mm, and that of BP is 9.189 mm. It can be seen that the improved IPSO-BP algorithm model has great advantages over other traditional models in predicting single-point settlement.
C) The improved IPSO-BP hybrid prediction model verifies that it can predict the surface settlement law during the construction process in the ultrashallow buried large-section rectangular pipe jacking tunnel project, and the results are more accurate, which can provide new ideas for settlement precontrol of similar projects. Based on the support of engineering intelligent big data, the applicability and accuracy of the model can be further improved by expanding the data set to strengthen learning.

Because the improved IPSO-BP hybrid prediction model does not take into account all the factors affecting surface subsidence, it has certain limitations. However, the method proposed in this study has certain universality. As long as the index selection can be based on the actual situation of the project, it can also be applied to study the settlement of other pipe jacking. When the project is relatively complex and there are many indicators selected, dimension reduction work should be further carried out. The higher-dimensional data are projected into the low-dimensional space to maximize the retention of the information of the original data and finally predict the calculation. Our next research will use more popular and stable algorithms to apply to other studies to help other researchers interested in this knowledge field.

## Data Availability

The datasets used and/or analysed during the current study available from the corresponding author on reasonable request.

## References

[1] Zhang Q, Wu K, Cui S (2019). Surface settlement induced by subway tunnel construction based on modified peck formula. Geotech. Geol. Eng..

[2] Tang X, Liang J, Liu W (2021). Modification of peck formula to predict ground surface settlement of twin tunnels in low permeability soil. Adv. Civ. Eng..

[3] Heng C, Sun S, Zhou Z (2019). Prediction of surface settlement with ultra-shallow-burial and large rectangular cross-section urban underpass. KSCE J. Civ. Eng..

[4] Gao Y, Liu Y, Tang P (2022). Modification of peck formula to predict surface settlement of tunnel construction in water-rich sandy cobble strata and its program implementation. Sustainability.

[5] Do Ngoc A, Dias D, Dang TT (2021). A numerical investigation of the impact of shield machine’s operation parameters on the settlements above twin stacked tunnels: A case study of ho chi minh urban railway line 1. Vietnam J. Earth Sci..

[6] Taherynia MH, Aghda SMF, Ghazifard A (2013). Modeling of land subsidence in the south pars gas field (iran). Int. J. Geosci..

[7] Sheorey P, Loui J, Singh K (2000). Ground subsidence observations and a modified influence function method for complete subsidence prediction. Int. J. Rock Mech. Min. Sci..

[8] Li W-X, Liu L, Dai L-F (2010). Fuzzy probability measures (fpm) based non-symmetric membership function: Engineering examples of ground subsidence due to underground mining. Eng. Appl. Artif. Intell..

[9] Hood M, Ewy R, Riddle L (1983). Empirical methods of subsidence prediction: A case study from Illinois. J. Rock Mech. Min. Sci. Geomech..

[10] Wu G, Jia S, Wu B (2018). A discussion on analytical and numerical modelling of the land subsidence induced by coal seam gas extraction. Environ. Earth Sci..

[11] Najjar, Y. & Zaman, M. Numerical modeling of ground subsidence due to mining. In *Proceedings of the the 34th US Symposium on Rock Mechanics (USRMS)* (OnePetro, 1993).

[12] Melis M, Medina L, Rodríguez JM (2002). Prediction and analysis of subsidence induced by shield tunnelling in the madrid metro extension. Can. Geotech. J..

[13] Guo Q, Meng X, Li Y (2021). A prediction model for the surface residual subsidence in an abandoned goaf for sustainable development of resource-exhausted cities. J. Clean. Prod..

[14] Wei L, Yinping L, Chunhe Y (2016). A new method of surface subsidence prediction for natural gas storage cavern in bedded rock salts. Environmental Earth Sciences.

[15] Thongprapha T, Fuenkajorn K, Daemen J (2015). Study of surface subsidence above an underground opening using a trap door apparatus. Tunn. Undergr. Space Technol..

[16] Fokker, P. A. Subsidence prediction and inversion of subsidence data. In *Proceedings of the SPE/ISRM Rock Mechanics Conference* (OnePetro, 2022).

[17] Ding Q, Shao Z, Huang X (2021). Monitoring, analyzing and predicting urban surface subsidence: A case study of wuhan city, china. Int. J. Appl. Earth Obs. Geoinf..

[18] Jain AK, Mao J, Mohiuddin KM (1996). Artificial neural networks: A tutorial. Computer.

[19] Javad G, Narges T (2010). Application of artificial neural networks to the prediction of tunnel boring machine penetration rate. Min. Sci. Technol..

[20] Pourtaghi A, Lotfollahi-Yaghin M (2012). Wavenet ability assessment in comparison to ann for predicting the maximum surface settlement caused by tunneling. Tunn. Undergr. Space Technol..

[21] Khatami, S. A. *et al*. Artificial neural network analysis of twin tunnelling-induced ground settlements. In *Proceedings of the 2013 IEEE International Conference on Systems, Man, and Cybernetics* (IEEE, 2013).

[22] Koukoutas S, Sofianos A (2015). Settlements due to single and twin tube urban epb shield tunnelling. Geotech. Geol. Eng..

[23] Xu W, Cheng M, Xu X (2019). Deep learning method on deformation prediction for large-section tunnels. Symmetry.

[24] Sun S (2022). Shield tunneling parameters matching based on support vector machine and improved particle swarm optimization. Sci. Programm..

[25] Liu X, Wang Z, Wang Y (2022). Predicting variation of multipoint earth pressure in sealed chambers of shield tunneling machines based on hybrid deep learning. Autom. Constr..

[26] Ling X, Kong X, Tang L (2022). Predicting earth pressure balance (epb) shield tunneling-induced ground settlement in compound strata using random forest. Transp. Geotech..

[27] Feng Z, Chen H, Zeng T (2022). Shield construction multiobjective optimization of surface settlement safety control based on machine learning. J. Phys..

[28] Chen J, Shen X, Chen Q (2022). Prediction of maximum surface settlements of bai∼ hua tunnel section based on machine learning. J. Phys..

[29] Ramezanshirazi, M., Sebastiani, D. & Miliziano, S. Artificial intelligence to predict maximum surface settlements induced by mechanized tunnelling. In *Proceedings of the National Conference of the Researchers of Geotechnical Engineering* (Springer, 2019).

[30] Tang JC, Peng L, Chen Z (2022). A computational approach of displacement prediction in an engineering project. J. Phys..

[31] Chen R, Zhang P, Wu H (2019). Prediction of shield tunneling-induced ground settlement using machine learning techniques. Front. Struct. Civ. Eng..

[32] Elbaz K, Shen S-L, Sun W-J (2020). Prediction model of shield performance during tunneling via incorporating improved particle swarm optimization into anfis. IEEE Access.

[33] Cao Y, Zhou X, Yan K (2021). Deep learning neural network model for tunnel ground surface settlement prediction based on sensor data. Math. Probl. Eng..

[34] Jin, G., Feng, W. & Meng, Q. Prediction of port container throughput based on pso optimization bp neural network model. In *Proceedings of the Proceedings of the 8th International Conference on Industrial and Business Engineering* (2022).

[35] Wan C, Yang J, Zhou L (2022). Fertilization control system research in orchard based on the pso-bp-pid control algorithm. Machines.

[36] Shu Y (2022). Research on customer perceived value evaluation of new chinese-style clothing based on pso-bp neural network. Sci. Programm..

